# Bis{2-[(*E*)-(4-fluoro­benz­yl)imino­meth­yl]-6-meth­oxy­phenolato}palladium(II)

**DOI:** 10.1107/S1600536811017739

**Published:** 2011-05-20

**Authors:** Hadariah Bahron, Amalina Mohd Tajuddin, Wan Nazihah Wan Ibrahim, Madhukar Hemamalini, Hoong-Kun Fun

**Affiliations:** a Faculty of Applied Sciences, Universiti Teknologi MARA, 40450 Shah Alam, Selangor, Malaysia; b X-ray Crystallography Unit, School of Physics, Universiti Sains Malaysia, 11800 USM, Penang, Malaysia

## Abstract

In the title compound, [Pd(C_15_H_13_FNO_2_)_2_], the Pd^II^ atom is tetra­coordinated by two N atoms and two O atoms from the two 2-[(4-fluoro­benz­yl)imino­meth­yl]-6-meth­oxy­phen­oxy ligands, forming a square-planar geometry. The two N atoms and the two O atoms around the Pd^II^ atom are *trans* to each other. The dihedral angle between the two fluoro-substituted benzene rings is 39.03 (6)°. The mol­ecular structure is stabilized by an intra­molecular C—H⋯O hydrogen bond. In the crystal, weak inter­molecular C—H⋯π inter­actions occur.

## Related literature

For applications of palladium(II)–Schiff base complexes, see: Ali *et al.* (2002 ▶); Gupta & Sutar (2008 ▶). For related structures, see: Jiang *et al.* (2008 ▶); Tsai *et al.* (2009 ▶); Mohd Tajuddin *et al.* (2010 ▶); Lin *et al.* (2010 ▶). For the stability of the temperature controller used in the data collection, see: Cosier & Glazer (1986 ▶).
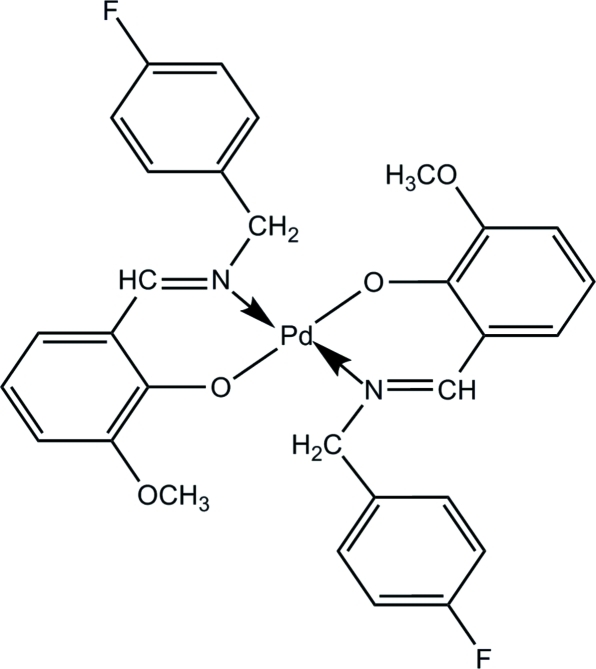


## Experimental

### 

#### Crystal data

[Pd(C_15_H_13_FNO_2_)_2_]
*M* *_r_* = 622.93
Triclinic, 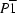

*a* = 10.0025 (4) Å
*b* = 11.0082 (4) Å
*c* = 12.3152 (4) Å
α = 109.550 (1)°
β = 98.368 (1)°
γ = 90.054 (1)°
*V* = 1262.45 (8) Å^3^
*Z* = 2
Mo *K*α radiation
μ = 0.79 mm^−1^
*T* = 100 K
0.54 × 0.19 × 0.16 mm

#### Data collection

Bruker SMART APEXII CCD area-detector diffractometer
Absorption correction: multi-scan (*SADABS*; Bruker, 2009 ▶) *T*_min_ = 0.677, *T*_max_ = 0.885
39095 measured reflections
11056 independent reflections
9774 reflections with *I* > 2σ(*I*)
*R*_int_ = 0.027

#### Refinement

*R*[*F* ^2^ > 2σ(*F* ^2^)] = 0.029
*wR*(*F* ^2^) = 0.081
*S* = 1.03
11056 reflections
354 parameters
H-atom parameters constrained
Δρ_max_ = 1.95 e Å^−3^
Δρ_min_ = −1.72 e Å^−3^

### 

Data collection: *APEX2* (Bruker, 2009 ▶); cell refinement: *SAINT* (Bruker, 2009 ▶); data reduction: *SAINT*; program(s) used to solve structure: *SHELXTL* (Sheldrick, 2008 ▶); program(s) used to refine structure: *SHELXTL*; molecular graphics: *SHELXTL*; software used to prepare material for publication: *SHELXTL* and *PLATON* (Spek, 2009 ▶).

## Supplementary Material

Crystal structure: contains datablocks global, I. DOI: 10.1107/S1600536811017739/is2710sup1.cif

Structure factors: contains datablocks I. DOI: 10.1107/S1600536811017739/is2710Isup2.hkl

Additional supplementary materials:  crystallographic information; 3D view; checkCIF report

## Figures and Tables

**Table 1:** Hydrogen-bond geometry (Å, °) *Cg*1, *Cg*2, *Cg*3 and *Cg*5 are the centroids of the Pd1/N1/O1/C1/C6/C7, Pd1/O2/N2/C16/C21/C22, C1–C6 and C16–C21 rings, respectively.

*D*—H⋯*A*	*D*—H	H⋯*A*	*D*⋯*A*	*D*—H⋯*A*
C29—H29*A*⋯O1	0.95	2.45	3.2051 (15)	136
C8—H8*B*⋯*Cg*2^i^	0.99	2.67	3.3882 (13)	130
C15—H15*B*⋯*Cg*5^ii^	0.98	2.71	3.6530 (15)	162
C23—H23*A*⋯*Cg*1^ii^	0.99	2.67	3.3612 (13)	127
C30—H30*C*⋯*Cg*3^i^	0.98	2.69	3.6146 (16)	158

Symmetry codes: (i) 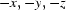; (ii) 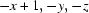.

**Fig. 1.**
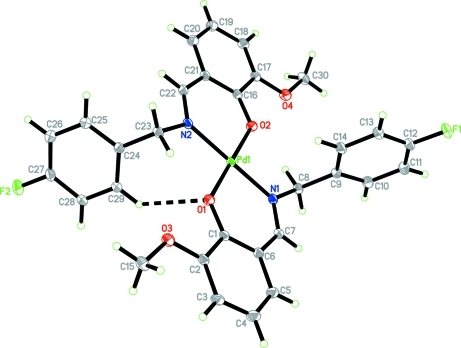
The molecular structure of the title compound, showing 30% probability displacement ellipsoids and the atom-numbering scheme.

**Fig. 2.**
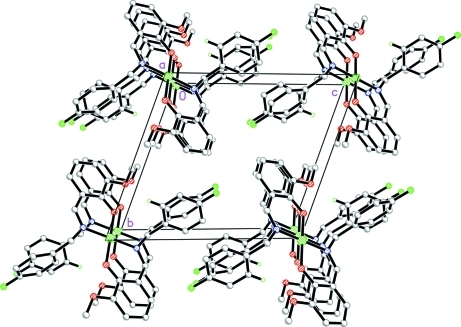
A crystal packing diagram of the title compound, viewed along the a axis.

## supplementary crystallographic information

### Comment

Palladium(II)-Schiff base complexes are well-known for their catalytic (Gupta & Sutar, 2008) and biological properties (Ali *et al.*, 2002). Schiff bases containing iminoalkylphenolato groups commonly perform bidentate coordination with metal centres as shown in bis{2-[(*E*)-benzyliminomethyl]-4,6-dibromophenalato-κ^2^N,O}cobalt(II) (Jiang *et al.*, 2008) and bis[2-(2*H*-benzotriazol-2-yl)-4-methyl-phenolato]palladium(II) (Tsai *et al.*, 2009). The title compound, (I), is bis-bidentate (see Fig. 1) and related to the previously reported bis[2-(1-benzyliminoethyl)phenolato]palladium(II) (Mohd Tajuddin *et al.*, 2010) but with different substituents on the iminoalkylphenolato and benzyl moieties.

The geometry around Pd^II^ atom is tetra-coordinated with a normal square planar environment in which two N atoms and two O atoms are coplanar. The two N atoms and two O atoms around the Pd^II^ atom are *trans* to each other.  The bond angles of O1—Pd1—N1= 92.57 (4)°, O2—Pd1—N1 = 87.15 (4)°, O1—Pd1—N2 = 87.59 (4)°, O2—Pd1—N2= 92.70 (4)°, N1—Pd1—N2 = 179.85 ° and O1—Pd1—O2 = 179.60 (3) °. The distances between the Pd^II^ atom and O and N are 1.9717 (9), 1.9727 (9), 2.0194 (10) and 2.0209 (10) Å, respectively. These bond distances and angles are similar to those found in the crystal structure of bis[4-methyl-2- (2*H*-benzotriazol-2-yl)phenolato]palladium (II) (Tsai *et al.*, 2009; Lin *et al.*, 2010).  The dihedral angle between the two fluoro-substituted benzene (C9–C14)/(C24–C29) rings is 39.03 (6)°.

In the crystal structure, (Fig. 2), the molecular packing is stablized by weak C29—H29A···O1 hydrogen bond and C—H···π interactions (Table 1), involving  centroids, *Cg*1 (Pd1/N1/O1/C1/C6/C7), *Cg*2 (Pd1/O2/N2/C16/C21/C22), *Cg*3 (C1–C6) and *Cg*5 (C16–C21).

### Experimental

(*E*)-2-[(4-fluorobenzyl)iminomethyl]-6-methoxyphenol (0.5196 g, 2 mmol) and palladium acetate (0.2249 g, 2 mmol) was each dissolved separately in acetonitrile (5 ml). The two solutions were then mixed and stirred under reflux for 4 hours upon which a brown precipitate was formed. It was isolated by gravity filtration, washed with cold acetonitrile and air dried at room temperature. The solid product was recrystallized from chloroform yielding yellow crystals (yield 97.1%, m.p. 526–529 K). Analytical calculation for C_30_H_26_F_2_N_2_O_4_Pd (%): C 57.84, H 4.21, N 4.50. Found (%): C 57.86, H 4.21, N 4.27. IR (cm^-1^): ν(C═N) 1616 (s), ν(C–O) 1248 (s), ν(C–H) 2836 (w), ν(C–N) 1328 (m), ν(C–OCH_3_) 1084 (m), ν(Pd–O) 581 (w), ν(Pd–N) 495 (w). ^1^H NMR (CDCl_3_, 300 MHz, p.p.m.): δ = 7.728 (1H, s, HC═N), 7.450–6.492

### Refinement

All hydrogen atoms were positioned geometrically (C—H = 0.95–0.99 Å) and were refined using a riding model, with *U*_iso_(H) = 1.2 or 1.5*U*_eq_(C). A rotating group model was applied to the methyl groups. There exists a pseudo-symmetry relation in the molecule that is broken by the deviating orientation of the fluorophenyl rings. The highest residual electron density peak and the deepest hole are located 0.68 and 0.65 Å, respectively, from atom Pd1.

### Crystal data

**Table:**

[Pd(C_15_H_13_FNO_2_)_2_]	*Z* = 2
*M**_r_* = 622.93	*F*(000) = 632
Triclinic, *P*1	*D*_x_ = 1.639 Mg m^−^^3^
Hall symbol: -P 1	Mo *K*α radiation, λ = 0.71073 Å
*a* = 10.0025 (4) Å	Cell parameters from 9774 reflections
*b* = 11.0082 (4) Å	θ = 2.5–35.1°
*c* = 12.3152 (4) Å	µ = 0.79 mm^−^^1^
α = 109.550 (1)°	*T* = 100 K
β = 98.368 (1)°	Block, yellow
γ = 90.054 (1)°	0.54 × 0.19 × 0.16 mm
*V* = 1262.45 (8) Å^3^	

### Data collection

**Table:**

Bruker SMART APEXII CCD area-detector diffractometer	11056 independent reflections
Radiation source: fine-focus sealed tube	9774 reflections with *I* > 2σ(*I*)
graphite	*R*_int_ = 0.027
φ and ω scans	θ_max_ = 35.2°, θ_min_ = 1.8°
Absorption correction: multi-scan (*SADABS*; Bruker, 2009)	*h* = −15→16
*T*_min_ = 0.677, *T*_max_ = 0.885	*k* = −17→17
39095 measured reflections	*l* = −19→19

### Refinement

**Table:**

Refinement on *F*^2^	Primary atom site location: structure-invariant direct methods
Least-squares matrix: full	Secondary atom site location: difference Fourier map
*R*[*F*^2^ > 2σ(*F*^2^)] = 0.029	Hydrogen site location: inferred from neighbouring sites
*wR*(*F*^2^) = 0.081	H-atom parameters constrained
*S* = 1.03	*w* = 1/[σ^2^(*F*_o_^2^) + (0.0422*P*)^2^ + 0.7245*P*] where *P* = (*F*_o_^2^ + 2*F*_c_^2^)/3
11056 reflections	(Δ/σ)_max_ = 0.001
354 parameters	Δρ_max_ = 1.95 e Å^−^^3^
0 restraints	Δρ_min_ = −1.72 e Å^−^^3^

### Special details

**Table:**

Experimental. The crystal was placed in the cold stream of an Oxford Cryosystems Cobra open-flow nitrogen cryostat (Cosier & Glazer, 1986) operating at 100.0 (1) K.
Geometry. All s.u.'s (except the s.u. in the dihedral angle between two l.s. planes) are estimated using the full covariance matrix. The cell s.u.'s are taken into account individually in the estimation of s.u.'s in distances, angles and torsion angles; correlations between s.u.'s in cell parameters are only used when they are defined by crystal symmetry. An approximate (isotropic) treatment of cell s.u.'s is used for estimating s.u.'s involving l.s. planes.
Refinement. Refinement of F^2^ against ALL reflections. The weighted R-factor wR and goodness of fit S are based on F^2^, conventional R-factors R are based on F, with F set to zero for negative F^2^. The threshold expression of F^2^ > 2σ(F^2^) is used only for calculating R-factors(gt) etc. and is not relevant to the choice of reflections for refinement. R-factors based on F^2^ are statistically about twice as large as those based on F, and R- factors based on ALL data will be even larger.

### Fractional atomic coordinates and isotropic or equivalent isotropic displacement parameters (Å^2^)

**Table:**

	*x*	*y*	*z*	*U*_iso_*/*U*_eq_	
Pd1	0.248588 (8)	−0.003817 (7)	−0.004344 (7)	0.01066 (3)	
F1	0.27490 (11)	−0.29889 (9)	0.45715 (8)	0.02764 (19)	
F2	0.20856 (10)	0.27200 (9)	−0.47652 (8)	0.02562 (18)	
O1	0.36986 (10)	0.15058 (9)	0.03227 (8)	0.01505 (15)	
O2	0.12766 (10)	−0.15832 (9)	−0.03987 (8)	0.01526 (16)	
O3	0.54754 (10)	0.32234 (9)	0.03930 (9)	0.01810 (17)	
O4	−0.04950 (10)	−0.33148 (9)	−0.04619 (9)	0.01818 (17)	
N1	0.17806 (10)	0.06583 (10)	0.14915 (9)	0.01244 (16)	
N2	0.31884 (10)	−0.07394 (10)	−0.15808 (9)	0.01247 (16)	
C1	0.39256 (12)	0.24727 (11)	0.13077 (10)	0.01246 (18)	
C2	0.49156 (12)	0.34497 (11)	0.13870 (10)	0.01365 (19)	
C3	0.52321 (13)	0.45087 (12)	0.23893 (11)	0.0169 (2)	
H3A	0.5896	0.5145	0.2426	0.020*	
C4	0.45737 (14)	0.46504 (13)	0.33603 (12)	0.0196 (2)	
H4A	0.4803	0.5375	0.4052	0.023*	
C5	0.36016 (14)	0.37416 (12)	0.33048 (11)	0.0175 (2)	
H5A	0.3150	0.3850	0.3956	0.021*	
C6	0.32634 (12)	0.26388 (11)	0.22825 (10)	0.01328 (18)	
C7	0.22130 (12)	0.17496 (11)	0.22910 (10)	0.01358 (18)	
H7A	0.1783	0.1991	0.2966	0.016*	
C8	0.07085 (12)	−0.00674 (11)	0.17704 (10)	0.01394 (19)	
H8A	0.0179	−0.0646	0.1035	0.017*	
H8B	0.0085	0.0549	0.2194	0.017*	
C9	0.12744 (12)	−0.08626 (11)	0.25037 (10)	0.01400 (19)	
C10	0.05718 (13)	−0.09792 (12)	0.33660 (11)	0.0178 (2)	
H10A	−0.0246	−0.0551	0.3486	0.021*	
C11	0.10491 (15)	−0.17143 (13)	0.40555 (12)	0.0207 (2)	
H11A	0.0562	−0.1801	0.4635	0.025*	
C12	0.22457 (15)	−0.23096 (12)	0.38736 (11)	0.0196 (2)	
C13	0.29778 (15)	−0.22301 (12)	0.30226 (11)	0.0196 (2)	
H13A	0.3797	−0.2658	0.2913	0.023*	
C14	0.24736 (13)	−0.15024 (12)	0.23327 (11)	0.0171 (2)	
H14A	0.2952	−0.1441	0.1739	0.021*	
C15	0.64506 (14)	0.41740 (13)	0.04021 (12)	0.0192 (2)	
H15A	0.6772	0.3917	−0.0354	0.029*	
H15B	0.7216	0.4250	0.1021	0.029*	
H15C	0.6034	0.5008	0.0546	0.029*	
C16	0.10422 (12)	−0.25480 (11)	−0.13811 (10)	0.01267 (18)	
C17	0.00590 (12)	−0.35324 (11)	−0.14546 (11)	0.01388 (19)	
C18	−0.02515 (13)	−0.45995 (12)	−0.24528 (11)	0.0170 (2)	
H18A	−0.0909	−0.5239	−0.2485	0.020*	
C19	0.04061 (15)	−0.47427 (12)	−0.34260 (12)	0.0191 (2)	
H19A	0.0184	−0.5473	−0.4114	0.023*	
C20	0.13651 (14)	−0.38259 (12)	−0.33769 (11)	0.0175 (2)	
H20A	0.1815	−0.3935	−0.4029	0.021*	
C21	0.16949 (12)	−0.27135 (11)	−0.23629 (10)	0.01365 (19)	
C22	0.27473 (12)	−0.18294 (11)	−0.23782 (10)	0.01387 (19)	
H22A	0.3171	−0.2074	−0.3056	0.017*	
C23	0.42827 (12)	−0.00312 (11)	−0.18626 (10)	0.01336 (18)	
H23A	0.4913	−0.0655	−0.2266	0.016*	
H23B	0.4799	0.0567	−0.1130	0.016*	
C24	0.37152 (12)	0.07264 (11)	−0.26307 (10)	0.01318 (18)	
C25	0.37981 (13)	0.02717 (12)	−0.38181 (10)	0.0167 (2)	
H25A	0.4228	−0.0510	−0.4136	0.020*	
C26	0.32631 (14)	0.09414 (13)	−0.45479 (11)	0.0190 (2)	
H26A	0.3325	0.0630	−0.5356	0.023*	
C27	0.26401 (14)	0.20716 (13)	−0.40591 (11)	0.0173 (2)	
C28	0.25358 (14)	0.25643 (12)	−0.28829 (11)	0.0173 (2)	
H28A	0.2099	0.3344	−0.2573	0.021*	
C29	0.30892 (13)	0.18839 (11)	−0.21691 (10)	0.0158 (2)	
H29A	0.3041	0.2210	−0.1358	0.019*	
C30	−0.14487 (14)	−0.42819 (13)	−0.04730 (13)	0.0194 (2)	
H30A	−0.1760	−0.4041	0.0286	0.029*	
H30B	−0.1019	−0.5110	−0.0629	0.029*	
H30C	−0.2223	−0.4360	−0.1084	0.029*	

### Atomic displacement parameters (Å^2^)

**Table:**

	*U*^11^	*U*^22^	*U*^33^	*U*^12^	*U*^13^	*U*^23^
Pd1	0.01183 (4)	0.00891 (4)	0.01172 (4)	−0.00181 (3)	0.00113 (3)	0.00440 (3)
F1	0.0435 (6)	0.0223 (4)	0.0244 (4)	0.0072 (4)	0.0073 (4)	0.0166 (3)
F2	0.0345 (5)	0.0268 (4)	0.0219 (4)	0.0067 (4)	0.0042 (3)	0.0165 (3)
O1	0.0188 (4)	0.0115 (3)	0.0142 (4)	−0.0055 (3)	0.0029 (3)	0.0033 (3)
O2	0.0182 (4)	0.0113 (3)	0.0152 (4)	−0.0052 (3)	0.0033 (3)	0.0028 (3)
O3	0.0202 (4)	0.0147 (4)	0.0195 (4)	−0.0066 (3)	0.0049 (3)	0.0052 (3)
O4	0.0190 (4)	0.0145 (4)	0.0217 (4)	−0.0050 (3)	0.0063 (3)	0.0058 (3)
N1	0.0132 (4)	0.0117 (4)	0.0136 (4)	−0.0011 (3)	0.0019 (3)	0.0059 (3)
N2	0.0137 (4)	0.0114 (4)	0.0137 (4)	−0.0011 (3)	0.0023 (3)	0.0059 (3)
C1	0.0134 (4)	0.0099 (4)	0.0144 (4)	−0.0013 (3)	0.0005 (3)	0.0052 (3)
C2	0.0144 (5)	0.0110 (4)	0.0158 (5)	−0.0023 (3)	0.0011 (4)	0.0055 (4)
C3	0.0175 (5)	0.0113 (4)	0.0200 (5)	−0.0038 (4)	0.0007 (4)	0.0035 (4)
C4	0.0223 (6)	0.0140 (5)	0.0191 (5)	−0.0034 (4)	0.0022 (4)	0.0018 (4)
C5	0.0211 (5)	0.0134 (5)	0.0163 (5)	−0.0024 (4)	0.0032 (4)	0.0028 (4)
C6	0.0147 (5)	0.0114 (4)	0.0138 (4)	−0.0014 (3)	0.0013 (3)	0.0047 (4)
C7	0.0155 (5)	0.0120 (4)	0.0143 (4)	−0.0002 (4)	0.0025 (4)	0.0057 (4)
C8	0.0133 (5)	0.0142 (4)	0.0156 (5)	−0.0016 (4)	0.0029 (4)	0.0064 (4)
C9	0.0161 (5)	0.0113 (4)	0.0148 (5)	−0.0022 (3)	0.0026 (4)	0.0047 (4)
C10	0.0202 (5)	0.0165 (5)	0.0189 (5)	−0.0015 (4)	0.0063 (4)	0.0075 (4)
C11	0.0266 (6)	0.0185 (5)	0.0206 (5)	−0.0024 (4)	0.0072 (5)	0.0096 (4)
C12	0.0301 (7)	0.0138 (5)	0.0174 (5)	−0.0005 (4)	0.0042 (4)	0.0085 (4)
C13	0.0256 (6)	0.0164 (5)	0.0196 (5)	0.0040 (4)	0.0053 (4)	0.0091 (4)
C14	0.0211 (5)	0.0150 (5)	0.0183 (5)	0.0018 (4)	0.0062 (4)	0.0084 (4)
C15	0.0179 (5)	0.0168 (5)	0.0245 (6)	−0.0048 (4)	0.0038 (4)	0.0090 (4)
C16	0.0130 (4)	0.0102 (4)	0.0153 (5)	−0.0014 (3)	0.0012 (3)	0.0053 (4)
C17	0.0141 (5)	0.0108 (4)	0.0169 (5)	−0.0020 (3)	0.0015 (4)	0.0053 (4)
C18	0.0180 (5)	0.0117 (4)	0.0196 (5)	−0.0042 (4)	−0.0001 (4)	0.0044 (4)
C19	0.0243 (6)	0.0126 (5)	0.0175 (5)	−0.0044 (4)	0.0013 (4)	0.0022 (4)
C20	0.0216 (5)	0.0131 (5)	0.0159 (5)	−0.0026 (4)	0.0022 (4)	0.0028 (4)
C21	0.0155 (5)	0.0114 (4)	0.0145 (5)	−0.0012 (3)	0.0018 (4)	0.0051 (4)
C22	0.0164 (5)	0.0121 (4)	0.0144 (5)	−0.0008 (4)	0.0033 (4)	0.0057 (4)
C23	0.0131 (4)	0.0121 (4)	0.0163 (5)	−0.0008 (3)	0.0034 (4)	0.0063 (4)
C24	0.0138 (4)	0.0121 (4)	0.0143 (4)	−0.0018 (3)	0.0027 (3)	0.0051 (4)
C25	0.0203 (5)	0.0149 (5)	0.0155 (5)	0.0013 (4)	0.0042 (4)	0.0052 (4)
C26	0.0235 (6)	0.0196 (5)	0.0146 (5)	0.0012 (4)	0.0039 (4)	0.0063 (4)
C27	0.0201 (5)	0.0180 (5)	0.0162 (5)	−0.0002 (4)	0.0015 (4)	0.0095 (4)
C28	0.0243 (6)	0.0125 (4)	0.0161 (5)	0.0001 (4)	0.0033 (4)	0.0059 (4)
C29	0.0219 (5)	0.0120 (4)	0.0141 (5)	0.0008 (4)	0.0037 (4)	0.0047 (4)
C30	0.0168 (5)	0.0165 (5)	0.0286 (6)	−0.0030 (4)	0.0051 (4)	0.0115 (5)

### Geometric parameters (Å, °)

**Table:**

Pd1—O1	1.9717 (9)	C11—H11A	0.9500
Pd1—O2	1.9727 (9)	C12—C13	1.3882 (19)
Pd1—N1	2.0194 (10)	C13—C14	1.3962 (18)
Pd1—N2	2.0209 (10)	C13—H13A	0.9500
F1—C12	1.3624 (16)	C14—H14A	0.9500
F2—C27	1.3599 (15)	C15—H15A	0.9800
O1—C1	1.3081 (14)	C15—H15B	0.9800
O2—C16	1.3053 (14)	C15—H15C	0.9800
O3—C2	1.3674 (15)	C16—C21	1.4156 (16)
O3—C15	1.4261 (15)	C16—C17	1.4339 (16)
O4—C17	1.3639 (15)	C17—C18	1.3818 (17)
O4—C30	1.4241 (16)	C18—C19	1.4132 (19)
N1—C7	1.2974 (15)	C18—H18A	0.9500
N1—C8	1.4817 (15)	C19—C20	1.3721 (18)
N2—C22	1.2974 (15)	C19—H19A	0.9500
N2—C23	1.4888 (15)	C20—C21	1.4225 (17)
C1—C6	1.4136 (16)	C20—H20A	0.9500
C1—C2	1.4305 (16)	C21—C22	1.4394 (17)
C2—C3	1.3804 (17)	C22—H22A	0.9500
C3—C4	1.4117 (19)	C23—C24	1.5117 (17)
C3—H3A	0.9500	C23—H23A	0.9900
C4—C5	1.3725 (18)	C23—H23B	0.9900
C4—H4A	0.9500	C24—C25	1.3934 (16)
C5—C6	1.4232 (17)	C24—C29	1.3996 (17)
C5—H5A	0.9500	C25—C26	1.3934 (18)
C6—C7	1.4388 (17)	C25—H25A	0.9500
C7—H7A	0.9500	C26—C27	1.3813 (18)
C8—C9	1.5111 (18)	C26—H26A	0.9500
C8—H8A	0.9900	C27—C28	1.3862 (17)
C8—H8B	0.9900	C28—C29	1.3929 (18)
C9—C10	1.3951 (17)	C28—H28A	0.9500
C9—C14	1.3983 (17)	C29—H29A	0.9500
C10—C11	1.3951 (19)	C30—H30A	0.9800
C10—H10A	0.9500	C30—H30B	0.9800
C11—C12	1.376 (2)	C30—H30C	0.9800
			
O1—Pd1—O2	179.60 (3)	C13—C14—H14A	119.6
O1—Pd1—N1	92.57 (4)	C9—C14—H14A	119.6
O2—Pd1—N1	87.15 (4)	O3—C15—H15A	109.5
O1—Pd1—N2	87.59 (4)	O3—C15—H15B	109.5
O2—Pd1—N2	92.70 (4)	H15A—C15—H15B	109.5
N1—Pd1—N2	179.85 (4)	O3—C15—H15C	109.5
C1—O1—Pd1	126.95 (8)	H15A—C15—H15C	109.5
C16—O2—Pd1	126.89 (8)	H15B—C15—H15C	109.5
C2—O3—C15	116.63 (10)	O2—C16—C21	125.54 (10)
C17—O4—C30	116.34 (10)	O2—C16—C17	116.76 (10)
C7—N1—C8	115.47 (10)	C21—C16—C17	117.69 (10)
C7—N1—Pd1	123.53 (8)	O4—C17—C18	124.87 (11)
C8—N1—Pd1	121.00 (7)	O4—C17—C16	113.91 (10)
C22—N2—C23	115.28 (10)	C18—C17—C16	121.21 (11)
C22—N2—Pd1	123.34 (8)	C17—C18—C19	120.21 (11)
C23—N2—Pd1	121.36 (7)	C17—C18—H18A	119.9
O1—C1—C6	125.61 (10)	C19—C18—H18A	119.9
O1—C1—C2	116.59 (10)	C20—C19—C18	119.96 (11)
C6—C1—C2	117.80 (10)	C20—C19—H19A	120.0
O3—C2—C3	125.21 (11)	C18—C19—H19A	120.0
O3—C2—C1	113.62 (10)	C19—C20—C21	120.91 (12)
C3—C2—C1	121.16 (11)	C19—C20—H20A	119.5
C2—C3—C4	120.27 (11)	C21—C20—H20A	119.5
C2—C3—H3A	119.9	C16—C21—C20	120.01 (11)
C4—C3—H3A	119.9	C16—C21—C22	123.08 (10)
C5—C4—C3	120.00 (12)	C20—C21—C22	116.84 (11)
C5—C4—H4A	120.0	N2—C22—C21	128.03 (11)
C3—C4—H4A	120.0	N2—C22—H22A	116.0
C4—C5—C6	120.70 (12)	C21—C22—H22A	116.0
C4—C5—H5A	119.7	N2—C23—C24	111.43 (10)
C6—C5—H5A	119.7	N2—C23—H23A	109.3
C1—C6—C5	120.05 (11)	C24—C23—H23A	109.3
C1—C6—C7	122.95 (10)	N2—C23—H23B	109.3
C5—C6—C7	116.98 (11)	C24—C23—H23B	109.3
N1—C7—C6	128.04 (11)	H23A—C23—H23B	108.0
N1—C7—H7A	116.0	C25—C24—C29	118.91 (11)
C6—C7—H7A	116.0	C25—C24—C23	120.18 (10)
N1—C8—C9	112.50 (10)	C29—C24—C23	120.91 (10)
N1—C8—H8A	109.1	C24—C25—C26	121.27 (11)
C9—C8—H8A	109.1	C24—C25—H25A	119.4
N1—C8—H8B	109.1	C26—C25—H25A	119.4
C9—C8—H8B	109.1	C27—C26—C25	117.94 (11)
H8A—C8—H8B	107.8	C27—C26—H26A	121.0
C10—C9—C14	118.88 (12)	C25—C26—H26A	121.0
C10—C9—C8	119.18 (11)	F2—C27—C26	118.60 (11)
C14—C9—C8	121.93 (10)	F2—C27—C28	118.47 (12)
C9—C10—C11	121.15 (12)	C26—C27—C28	122.92 (12)
C9—C10—H10A	119.4	C27—C28—C29	118.09 (11)
C11—C10—H10A	119.4	C27—C28—H28A	121.0
C12—C11—C10	118.16 (12)	C29—C28—H28A	121.0
C12—C11—H11A	120.9	C28—C29—C24	120.88 (11)
C10—C11—H11A	120.9	C28—C29—H29A	119.6
F1—C12—C11	118.70 (12)	C24—C29—H29A	119.6
F1—C12—C13	118.39 (13)	O4—C30—H30A	109.5
C11—C12—C13	122.90 (13)	O4—C30—H30B	109.5
C12—C13—C14	118.01 (12)	H30A—C30—H30B	109.5
C12—C13—H13A	121.0	O4—C30—H30C	109.5
C14—C13—H13A	121.0	H30A—C30—H30C	109.5
C13—C14—C9	120.89 (11)	H30B—C30—H30C	109.5

### Hydrogen-bond geometry (Å, °)

**Table:**

*Cg*1, *Cg*2, *Cg*3 and *Cg*5 are the centroids of the Pd1/N1/O1/C1/C6/C7, Pd1/O2/N2/C16/C21/C22, C1–C6 and C16–C21 rings, respectively.

**Table:**

*D*—H···*A*	*D*—H	H···*A*	*D*···*A*	*D*—H···*A*
C29—H29A···O1	0.95	2.45	3.2051 (15)	136
C8—H8B···Cg2^i^	0.99	2.67	3.3882 (13)	130
C15—H15B···Cg5^ii^	0.98	2.71	3.6530 (15)	162
C23—H23A···Cg1^ii^	0.99	2.67	3.3612 (13)	127
C30—H30C···Cg3^i^	0.98	2.69	3.6146 (16)	158

Symmetry codes: (i) −*x*, −*y*, −*z*; (ii) −*x*+1, −*y*, −*z*.
